# Factors Related to Depression According to the Degree of Loneliness in Adolescents with Severe Friend-Relationship Stress

**DOI:** 10.3390/healthcare11091354

**Published:** 2023-05-08

**Authors:** Hyeok Gyu Park, Sunkyung Jeong, Myoungjin Kwon

**Affiliations:** 1 Department of Nursing, Pai Chai University, Daejeon 35345, Republic of Korea; 2 Department of Nursing, Chung Cheong University, Cheongju-si 28171, Republic of Korea; 3 Department of Nursing, Daejeon University, Daejeon 34520, Republic of Korea

**Keywords:** depression, loneliness, adolescents, stress, friend relationship

## Abstract

This study attempted to identify factors affecting depression in relation to loneliness among adolescents. The study participants were 2668 adolescents aged 12 to 18 years who felt stressed and lonely. The variables in this study were classified into sociodemographic and psychological characteristics based on the 16th Korea Youth Risk Behavior Web-Based Survey items. Results revealed that sex, smoking, suicidal ideation, suicide planning, and anxiety were significantly related to depression (*p* < 0.05) in the group that reported less loneliness. Economic level, alcohol consumption, suicidal ideation, suicide planning, subjective body type, and anxiety were significantly related factors (*p* < 0.05) in the group that reported feeling very lonely. This study is significant in providing basic data for developing evidence-based strategies to ameliorate depression in adolescents.

## 1. Introduction

The influence of friends in adolescence can be considered to be very high because adolescents experience rapid changes through cognitive, physical, and social development during this period and feel a sense of identity with friends who share the same experiences, increasing their dependence on peers. Adolescents form special social relationships called peer groups and can experience loneliness if they are not embraced as “we” in their groups, such as in their schools or the social identity that individuals give meaning to [1]. Loneliness is an unpleasant and painful emotion characterized by feelings of emptiness [2], and frustration with the desire to interact socially and emotionally in community relationships based on social needs theory and cognitive perspectives can cause loneliness in adolescents who want to feel a sense of belonging [3]. Adolescents who have experienced being unrecognized or rejected in friendships can perceive friendships as a negative stressor and become predisposed to loneliness. The higher the perception of loneliness, the more difficult it is to adapt to daily life. Loneliness in adolescents can lead to intentions to drop out of school due to maladjustment to school life, being led astray, increased stress, depression, and suicidal ideation [4,5,6,7]. In addition, the use of smartphones to overcome the loneliness caused by a lack of relationships can cause addictions, such as smartphone addictions, and loneliness can also result in habitual drug use [8,9,10]. According to previous studies, addiction has negative consequences for loneliness and stress, and depression also increases, resulting in maladjustment to school life, which can lead to a vicious cycle [11].

According to the *2019 Youth Statistics* published by the Ministry of Gender Equality and Family and Statistics Korea, the rate of experiencing depression increased such that high school students had a higher rate of experiencing depression than middle school students, and the higher the grade, the higher the risk of depression [12]. As such, depression tends to increase during the transition stage from adolescence to adulthood, so it is necessary to confirm the actual situation and identify the related influencing factors. Depression is not a temporary mood state but a phenomenon in which depressed states such as sadness, loneliness, and emptiness appear continuously [13]. Higher levels of depression have been shown to be associated with greater difficulties in adapting to school life and interpersonal relationships [14]. In particular, adolescents experience more depression and anxiety when their peer relationships, which are an important part of their interpersonal relationships, are negative [15,16,17]. Among adolescents’ interpersonal interactions, it has been found that stress from friend relationships increases depression [18], and increased stress and depression in adolescents result in smartphone overdependence, social network services (SNS) addictions, video game addictions, and habitual drug use [5,10]. In addition, increased stress and depression in adolescents have been shown to increase suicidal ideation, which is a psychological problem. Loneliness is a major predictor of suicidal ideation in adolescents [19], and the relationship between depression, suicidal ideation, and loneliness has been discussed in several studies [5,15,19]. The factors that affect depression in adolescents include sociodemographic factors, home-environment-related factors, and educational-environment-related factors [20]. Sociodemographic factors, physical-health-related factors, and mental factors have also been explored as sub-variables affecting depression in adolescents. One variable that has been suggested to affect adolescents’ depression based on the ecological system theory is friendship [21]. Therefore, in this study, related factors that can affect depression according to the degree of loneliness related to friendship stress in adolescents are divided into three categories: sociodemographic factors, health-related factors, and psychological factors. Previous studies have shown a relationship between depression, suicidal ideation, and addiction. However, this study provides more useful and empirical basic data that can be individually accessed according to the degree of loneliness of the subjects by identifying the differences in depression, suicidal ideation, body image, smartphone addiction, and anxiety according to the degree of loneliness.

Thus, the purpose of this study is to identify the health problems of adolescents by exploring the factors affecting depression in adolescents due to loneliness and to provide basic data for early intervention measures for adolescents with depression. Therefore, this study will aim to provide basic data for early intervention plans for adolescents with health and depression problems by identifying the factors that affect depression due to loneliness, which is the main variable described in previous studies.

## 2. Materials and Methods

### 2.1. Research Design

This was a cross-sectional study with secondary data analysis to identify the factors related to depression in relation to the degree of loneliness in adolescents with friend-relationship stress.

### 2.2. Study Participants

The Korea Youth Risk Behavior Web-Based Survey (KYRBWS) is an annual survey conducted by the Ministry of Education, the Ministry of Health and Welfare, and the Korea Centers for Disease Control and Prevention for youth health policies and health promotion projects. This study obtained data for KYRBWS participants aged 12–18 years. Among the 54,948 participants in 2020, 2668 adolescents who responded to questions related to stress and loneliness were included in this study. The question on stress, “How much stress do you feel in your daily life?”, consisted of a 5-point scale with the options “I feel very much”, “I feel a lot”, “I feel a little”, “I do not feel much”, and “I do not feel it at all.”

The question about the cause of stress is “What is the biggest cause of stress?”. Subjects choose one option: conflicts with parents, family situations, conflicts with teachers, conflicts with friends, conflicts with seniors and juniors, grades, career, health problems, appearance, and others. Among the subjects who answered ‘I feel a lot’, ‘I feel a lot’, or ‘I feel a little’ of stress, 2668 people answered that the cause of their stress was friends and seniors and juniors. Loneliness was measured with the question, “In the past 12 months, how often have you felt lonely?” Subjects chose one option from the following choices: “I never felt lonely”, “I rarely felt lonely”, “I felt lonely sometimes”, “I felt lonely often”, or “I felt lonely all the time”. “I rarely felt lonely” and “Sometimes I felt lonely” were classified as feeling less lonely. “I often felt lonely” and “I felt lonely all the time” were classified as feeling lonely a lot. Among the 2668 participants, 1773 answered that they felt less lonely, and 895 answered that they felt very lonely.

### 2.3. Research Variables

The variables in this study were classified into sociodemographic, physical health, and psychological factors based on the KYRBWS items.

#### 2.3.1. Sociodemographic Factors

Sociodemographic factors included sex (male, female), age, academic grade (upper, middle, lower), economic level (upper, middle, lower), living with family (yes or no), experience of sexual intercourse (yes or no), and experience of treatment due to violence (yes or no).

#### 2.3.2. Physical Health Factors

Physical health factors consisted of smoking (yes, no), smoking amount per day (1 cigarette or less, 2–9 cigarettes, 10 cigarettes or more), alcohol consumption (yes, no), amount of alcohol consumed per drink (1–2 glasses of soju, 3–6 glasses of soju, more than 1 bottle of soju), the degree of fatigue recovery by sleep over the last 7 days (enough, not enough), and body mass index (BMI) (less than 18.5 kg/m^2^, 18.5–22.9 kg/m^2^, and 23 kg/m^2^ or more) [22].

#### 2.3.3. Psychological Factors

Psychological factors consisted of depression (yes, no), suicidal ideation (yes, no), suicide planning (yes, no), suicide attempt (yes, no), loneliness (feeling a little lonely, feeling very lonely), subjective body shape perception (underweight, normal, overweight), smartphone addiction (yes, no), and anxiety.

Smartphone addiction was measured by the smartphone dependence scale developed by the Ministry of Science, ICT and Future Planning, and the Korea Information Society Agency that integrated the existing Internet (K-scale) and smartphone (S-scale) individual scales [23]. This was a 4-point Likert scale with a total of 10 items, and the score ranged from 10 to 40. For adolescents, scores of 31 points or more corresponded to a high risk of smartphone addiction, and scores of 23–30 points corresponded to a potential risk of smartphone addiction. Therefore, in this study, the cutoff score for classifying smartphone addiction was 23 points. Anxiety was assessed using the Generalized Anxiety Disorder-7 (GAD-7) scale developed by Spitzer et al. [24]. The GAD-7 is a 4-point Likert scale with seven items and measures the frequency of symptom experience over the past two weeks. The scores range from 0 to 21, with higher scores indicating more severe anxiety.

### 2.4. Data Analysis

Analysis was performed after generating a complex sample plan file by assigning weights using IBM SPSS 25.0. The significance level was set at *p* < 0.05. To compare the two groups in relation to the degree of loneliness, a complex sample χ^2^-test and *t*-test were conducted. In addition, composite sample logistic regression analysis was performed to identify factors related to depression in the two groups. In the complex sample logistic regression analysis, the dependent variable was depression, and the independent variable was input as a significant variable in the χ^2^-test and *t*-test.

## 3. Results

### 3.1. Comparison of Sociodemographic Characteristics of Subjects

As shown in Table 1, the two groups categorized by feelings of loneliness showed significant differences in sociodemographic characteristics such as sex, age, economic level, experiences of sexual intercourse, and experiences of treatment due to violence (*p* < 0.05).

More women in the group that felt very lonely were older and had a lower economic status than those who felt less lonely. The group that felt very lonely had more experiences of sexual intercourse and treatment due to violence.

### 3.2. Comparison of Health-Related Characteristics of the Participants

As shown in Table 2, the two groups showed significant differences in health-related characteristics, such as smoking, drinking, and the degree of fatigue recovery by sleep over the last seven days (*p* < 0.05).

Participants who felt very lonely were more likely to smoke and drink, and those who felt less lonely were better at recovering from fatigue through sleep.

### 3.3. Comparison of Psychological Characteristics of Subjects 

As shown in Table 3, the two groups showed significant differences in terms of depression, suicidal ideation, suicide planning, suicide attempts, subjective body shape perception, smartphone addiction, and anxiety (*p* < 0.05).

Depression, suicidal thoughts, suicide plans, and suicide attempts were more common in the group that felt very lonely. The participants in the group that felt very lonely included more adolescents with underweight and obese subjective body shape perceptions and showed higher rates of smartphone addiction and anxiety.

### 3.4. Factors Related to Depression in the Participants

As shown in Table 4, logistic regression analysis was performed with the remaining variables, except for academic grade, living with family, daily smoking amount, alcohol consumption per drink, and BMI, which showed no significant differences in the χ^2^-test and *t*-test, as independent variables and depression as the dependent variable. As shown in Table 3, in the group that felt less lonely, sex, smoking, suicidal ideation, suicide planning, and anxiety were significantly associated with depression (*p* < 0.05).

Males were 0.54 times (95% CI: 0.41–0.71) less depressed than females, and non-smokers were 0.52 times (95% CI: 0.34–0.80) less depressed than smokers. Participants without suicidal thoughts and plans showed 0.21 times (95% CI: 0.14–0.34) and 0.34 times (95% CI: 0.12–0.94) less depression than participants with suicidal thoughts and plans. As anxiety increased, depression also increased 1.16 times (95% CI: 1.12–1.20).

In the group that felt very lonely, economic level, suicidal ideation, suicide planning, subjective body shape perception, and anxiety were significantly related (*p* < 0.05). Depression was 1.74 times higher among those with a higher economic status than among those with a lower economic status. Participants without suicidal thoughts and plans showed 0.52 times (95% CI: 0.34–0.81) and 0.19 times (95% CI: 0.07–0.54) less depression than participants with suicidal thoughts and plans. Participants with normal subjective body shape perception were 1.83 times (95% CI: 1.20–2.81) more depressed than obese participants. Depression was found to increase 1.17 times (95% CI: 1.11–1.22) as anxiety increased.

## 4. Discussion

This study attempted to explore the factors that affects loneliness and adolescent depression, identify adolescent health problems in detail, and provide basic data for early intervention in adolescent depression using raw data from the 2020 16th KYRBWS.

The results showed that female students felt more depressed than male students in the group that felt less lonely. This is consistent with the results of previous studies [25,26,27]. Women have role characteristics such as passivity and self-sacrifice, which can be explained by their gender role characteristics, and these role characteristics can lead to depression [25]. In contrast, in the group that felt very lonely, there was no significant difference in depression between the male and female students. For male students, the lower their attachment to peers, the higher their depression [27]. Thus, male students who felt very lonely did not show a significant difference from female students because their depression increased.

This study showed that adolescents who felt less lonely showed less depression in the nonsmoking group than in the smoking group. In the CHA study [27], students who had smoking experience showed higher levels of depression than non-smokers, supporting the results of this study. The repeated use of nicotine directly induces depression, which leads to nicotine addiction, and leads to smoking again to get rid of withdrawal symptoms that appear when blood nicotine levels drop [27]. These findings highlight the need for an adolescent smoking cessation program because adolescent smoking is not simply caused by curiosity but is also linked to psychological factors such as depression.

The results of this study showed that among the group that felt very lonely, depression was higher in the group with a high economic status than in the group with a low economic status. In the CHA study, students with a high economic status had lower levels of depression than those with a middle economic status, and students with a low economic status had higher levels of depression than those with a high economic status. In another previous study, students with low economic status had higher rates of depression than students with medium-to-high economic status [25]. Thus, the results linking the rates of depression with economic status appear to vary across studies, and further studies are required to identify the reasons underlying these variations.

Both groups felt more depressed in the group with suicidal ideation and plans, and depression increased with anxiety. Numerous previous studies have shown a positive correlation between depression and suicidal ideation and planning, with the results showing that higher rates of depression are associated with higher levels of suicidal ideation and planning as well as suicide attempts [28,29,30]. Adolescents often choose suicide as an extreme expression of depression, which is a strong predictor of suicide [29,31]. Therefore, both loneliness groups can be interpreted as showing significant results.

It is known in previous studies that smartphone addiction is a causative variable affecting depression [4,9], but this study did not show a significant difference. Therefore, it is considered necessary to conduct repeated studies or longitudinal studies on that variable.

Meanwhile, the frequency of depression was very high in the adolescent group with high anxiety, and the rate of the coexistence of depression and anxiety was very high [27]. Therefore, to effectively mediate depression in adolescents, both depression and anxiety should be assessed, and factors related to suicide should be examined together.

In this study, in the group that felt very lonely, the group with a normal subjective body shape perception felt more depressed than the obese group. This differs from the results of many previous studies [32,33], showing that subjective obesity forms a negative body image and leads to depression. Therefore, a more comprehensive study on subjective body shape perception and depression among adolescents is required.

As a result of this study, all psychological characteristics showed significant differences according to loneliness. Suicidal ideation, suicidal plans, and suicidal attempts were more common in the group that felt very lonely. This is consistent with the results of previous studies [6]. Loneliness is an important internal stimulating factor that causes suicidal ideation in adolescents and is considered a major predictor of suicidal ideation [6]. The prevention of suicidal ideation is very important because suicide is a continuum concept that starts with suicidal ideation and leads to suicidal plans and suicidal attempts. In particular, adolescence is a period of psychological immaturity, so impulsiveness is stronger than other periods [5,6]. Therefore, loneliness, which affects adolescents’ suicidal ideation, should not be easily overlooked, and it should be recognized that it requires a lot of attention and management. In order to prevent loneliness and suicide in adolescents, active strategies, including careful observation and individual counseling using psychosocial models, are required.

Adolescents are relatively less able to cope with loneliness than adults, which leads to negative consequences such as school maladaptation, stress, and depression. Recently, adolescents have tended to choose smartphones to overcome loneliness, which leads to smartphone addiction, and smartphone addiction increases stress and loneliness again [34]. In this study, it was found that the group that felt very lonely had a high rate of smartphone addiction, and since smartphone addiction negatively affects mental health, including loneliness in adolescents, it is necessary to minimize smartphone addiction.

The group that felt very lonely had higher levels of depression and anxiety. Anxiety in adolescents can lead to depression, and the longer the duration of anxiety disorder, the more suicidal ideation tends to increase. Adolescents are psychologically unstable and are sensitive to external stimuli and changes, and their reactions to them are also rougher and more impulsive than adults. Therefore, there is a high possibility that such anxiety will lead to suicide [11,31].

Anxiety in adolescents is accompanied by stress and depression, as well as suicide, and has a more negative impact on adolescent mental health [28]. Depression is the most dangerous factor leading to suicide, and there is also a study showing that more than 90% of adolescents who actually commit suicide have depressive symptoms [11,29]. As such, anxiety and depression have a very large impact on adolescent suicide and mental health and are related to loneliness. Therefore, in order to assess and manage the mental health of adolescents, strategies are needed to investigate and resolve these factors more closely.

As a result of this study, participants who felt very lonely were more likely to smoke and drink. Adolescents’ smoking and drinking have a negative impact not only on their physical health but also on their mental health, and they easily fall into misuse and abuse. In addition, since the nerve cells of adolescents have not matured, smoking and drinking cause damage to and functional decline in nerve cells, which can cause severe emotional instability or psychosis and negatively affect intelligence development and personality formation [35]. Frequent drinking in adolescence reduces academic achievement and tends to lead to problematic drinking, such as excessive drinking and drunkenness in adulthood. In addition, smoking and drinking can cause serious social problems as a catalyst for juvenile delinquency [35,36]. Because smoking and drinking in adolescents are related to mental health factors such as depression, happiness, and suicidal ideation, as well as loneliness, it is necessary to develop and apply preventive education and nursing intervention programs for adolescent smoking and drinking to verify their effectiveness and mental factors, including loneliness, that should be taken into account when developing programs.

Finally, in this study, the factors influencing depression and loneliness and their relationship in adolescents with severe friendship stress were identified, and it is thought that this will be important in developing strategies for improving adolescent mental health in the future.

## 5. Conclusions

In this secondary data analysis to identify factors related to depression in relation to the degree of loneliness in adolescents with friend-relationship stress, we found that the factors related to depression were sex, smoking, suicidal ideation, suicidal planning, anxiety, economic status, and subjective body type.

Therefore, to improve depression in adolescents, various causes of depression should be explored in more detail, and a multifaceted approach should be taken. In addition, suicidal ideation and suicidal planning are deeply related to depression in adolescents, so they must be considered when developing interventions to improve depression.

This study had the following limitations. First, this was a cross-sectional study; therefore, causality could not be identified, necessitating future longitudinal studies. Second, because the 2020 16th KYRBWS is a self-administered survey, it is based on a subjective perception of depression, and it is not able to accurately and deeply grasp the aspect of depression. It will be necessary to measure depression using an objective tool in the future.

Despite these limitations, this study has academic significance since it provides basic data for the development of evidence-based strategies for improving depression in adolescents.

## Figures and Tables

**Table 1:** Sociodemographic characteristics of the participants (*n* = 2668).

Characteristics		Feeling Less Lonely	Feeling Very Lonely	x^2^/t (*p*)
		*n* (Weight %)/Mean (SE)		
Sex	Male	761 (43.2)	298 (33.5)	23.79 (<0.001)
	Female	1012 (56.8)	597 (66.5)	
Age		14.92 (0.05)	15.16 (0.06)	−2.91 (0.004)
Academic grade	Upper	576 (32.4)	267 (30.2)	2.79 (0.307)
	Middle	562 (31.4)	265 (30.3)	
	Lower	635 (36.2)	363 (39.5)	
Economic level	Upper	625 (36.5)	293 (36.4)	14.69 (0.004)
	Middle	905 (50.1)	418 (44.8)	
	Lower	243 (13.4)	184 (18.8)	
Living with family	Yes	1626 (93.9)	813 (93.2)	0.50 (0.507)
	No	147 (6.1)	82 (6.8)	
Experience of sexual intercourse	Yes	115 (6.6)	92 (10.1)	9.60 (0.008)
	No	1658 (93.4)	803 (89.9)	
Experience of treatment due to violence	Yes	54 (3.1)	49 (6.0)	13.30 (0.002)
	No	1719 (96.9)	846 (94.0)	

**Table 2:** Health-related characteristics of the participants (*n* = 2668).

Characteristics		Feeling Less Lonely	Feeling Very Lonely	x^2^ (*p*)
		*n* (Weight %)		
Smoking	Yes	243 (14.2)	177 (19.6)	12.72 (0.002)
	No	1530 (85.8)	718 (80.4)	
Amount of smoking (cigarettes/day)	≤1	25 (20.2)	20 (25.96)	1.40 (0.565)
	2–9	75 (58.6)	42 (50.8)	
	≥10	30 (21.2)	24 (23.3)	
Drinking	Yes	676 (37.6)	436 (49.0)	32.17 (<0.001)
	No	1097 (62.4)	459 (51.0)	
Amount of alcohol consumed per drink (/glass)	1–2	92 (32.1)	59 (32.9)	0.34 (0.872)
	3–6	63 (23.5)	43 (25.4)	
	≥7	107 (44.4)	71 (41.7)	
Fatigue recovery by sleep	Much	483 (28.1)	143 (16.0)	50.32 (<0.001)
	A little	1290 (71.9)	752 (84.0)	
Body mass index (kg/m^2^)	<18.5	386 (23.2)	212 (25.6)	2.21 (0.409)
	18.5–22.9	885 (52.6)	433 (49.9)	
	≥23	411 (24.1)	215 (24.5)	

**Table 3:** Psychological characteristics of the participants (*n* = 2668).

Characteristics		Feeling Less Lonely	Feeling Very Lonely	x^2^/t (*p*)
		*n* (Weight %)/Mean (SE)		
Depression	Yes	570 (32.9)	639 (71.9)	375.29 (<0.001)
	No	1203 (67.1)	256 (28.1)	
Suicidal ideation	Yes	185 (10.3)	349 (39.3)	299.09 (<0.001)
	No	1588 (89.7)	546 (60.7)	
Suicidal planning	Yes	45 (2.5)	114 (13.5)	118.14 (<0.001)
	No	1728 (97.5)	781 (86.5)	
Suicide attempt	Yes	32 (1.7)	82 (8.9)	72.61 (<0.001)
	No	1741 (98.3)	813 (91.1)	
Subjective body shape perception	Underweight	447 (25.4)	262 (30.0)	10.59 (0.014)
	Normal	674 (38.2)	283 (32.3)	
	Overweight	652 (36.4)	350 (37.7)	
Smartphone addiction	Yes	495 (28.0)	378 (41.9)	51.69 (<0.001)
	No	1278 (72.0)	517 (58.1)	
Anxiety		4.34 (0.10)	9.74 (0.20)	−22.77 (<0.001)

**Table 4:** Factors related to depression in the participants (*n* = 2668).

Characteristics		Feeling Less Lonely		Feeling Very Lonely	
		OR	95% CI	OR	95% CI
Sex (female)	Male	0.54 **	0.41–0.71	0.70	0.47–1.04
Age		1.03	0.96–1.11	1.07	0.96–1.18
Economic level (lower)	Upper	1.11	0.76–1.61	1.74 *	1.04–2.90
	Middle	1.05	0.75–1.49	1.01	0.62–1.65
Sexual experience (yes)	No	0.85	0.52–1.40	0.69	0.32–1.50
Treatment experience due to violence (yes)	No	1.27	0.53–3.05	0.40	0.10–1.52
Smoking (yes)	No	0.52 *	0.34–0.80	0.80	0.46–1.38
Drinking (yes)	No	0.73	0.54–1.01	0.90	0.60–1.34
Fatigue recovery by sleep (a little)	Much	0.95	0.70–1.27	0.94	0.59–1.50
Suicidal ideation (yes)	No	0.21 **	0.14–0.34	0.52 *	0.34–0.81
Suicidal plan (yes)	No	0.34 *	0.12–0.94	0.19 *	0.07–0.54
Suicidal attempt (yes)	No	0.88	0.32–2.41	1.05	0.40–2.73
Subjective body shape perception (overweight)	Underweight	0.97	0.70–1.36	1.53	0.97–2.41
	Normal	0.92	0.68–1.25	1.83 *	1.20–2.81
Smartphone addiction (yes)	No	0.88	0.66–1.17	1.37	0.94–1.99
Anxiety		1.16 **	1.12–1.20	1.17 **	1.11–1.22

*: <0.05, **: <0.001.

## Data Availability

KYRBWS data are publicly accessible. The data can be accessed and downloaded from the KYRBWS homepage (URL: https://https://www.kdca.go.kr/yhs/, accessed on 10 October 2021).
